# Association of work ability with job burnout and sleep quality among biosafety laboratory personnel in Xinjiang, China: a cross-sectional study

**DOI:** 10.3389/fpubh.2024.1479257

**Published:** 2025-02-20

**Authors:** Keke Ju, Ruikai Wu, Jing Yu, Lei Ding, Mengjie Xia, Jiwen Liu, Yaoqin Lu

**Affiliations:** ^1^Shaanxi Provincial People’s Hospital, Xi'an, Shanxi, China; ^2^School of Public Health, Xinjiang Medical University, Ürümqi, China; ^3^The Scientific and Educational Department of the Health Commission of the Autonomous Region, Ürümqi, China; ^4^Urumqi City Center for Disease Control and Prevention, Ürümqi, China

**Keywords:** biosafety laboratory personnel, occupational burnout, sleep quality, work ability, relationship

## Abstract

**Background:**

In recent years, the importance of biosafety research has garnered significant attention due to its critical implications for public health and safety. Biosafety Laboratory (BSL) personnel face numerous challenges as they work with high-risk pathogens, including high-pressure environments, stringent safety protocols, and the risk of infection. Research indicates that occupational stress and burnout significantly affect the physical and mental well-being of laboratory personnel, potentially diminishing their work efficiency and capabilities. This study aims to investigate the effects of occupational burnout and sleep quality on the work ability of BSL personnel in Xinjiang, ultimately providing valuable insights for enhancing biosafety and improving work efficiency.

**Methods:**

In July 2022, a cluster sampling method was employed to survey the staff of BSL in Xinjiang. The study utilized the Maslach Burnout Inventory, the Pittsburgh Sleep Quality Index, and the Work Ability Index to assess the levels of occupational burnout, sleep quality, and work ability among the BSL personnel. Statistical analyses were performed using R Studio 4.2.2 and AMOS 26.0. Through t-tests, analysis of variance, and logistic regression analyses, the study explored the current status and influencing factors of work ability among Xinjiang BSL personnel, as well as the interactive and mediating effects of occupational burnout and sleep quality on work ability.

**Results:**

The prevalence of occupational burnout was 67.4%, while the prevalence of sleep disorders was 38.9%. Interaction analysis revealed that BSL personnel experiencing both occupational burnout and sleep disorders faced a risk of impaired work ability 21.43 times greater than those without burnout and with good sleep quality (*OR* = 21.43, 95%*CI*: 14.30–32.12). Structural equation modeling indicated that occupational burnout indirectly impacts work ability through its effect on sleep quality. The indirect effect was significant with a path coefficient of *β* = −0.28 (0.64 × −0.43), *p* < 0.01.

**Conclusion:**

The overall work ability of staff in BSL in Xinjiang is relatively good. Occupational burnout and sleep quality are significant risk factors affecting the work ability of BSL personnel. Improving occupational burnout and sleep quality can enhance the work ability of BSL personnel both directly and indirectly.

## Introduction

In recent years, biosafety research has garnered widespread attention as a critical area for safeguarding public health and safety. Biosafety Laboratory (BSL) serve as facilities for conducting experiments with highly pathogenic microorganisms (1). The primary functions of these laboratories include: pathogen research, which involves studying various pathogens (such as bacteria, viruses, fungi, and parasites) to understand their characteristics, pathogenic mechanisms, and modes of transmission (2); vaccine and drug development, aimed at creating new vaccines and treatments to prevent or cure diseases caused by these pathogen (3); and biomarker research, which focuses on discovering and validating biomarkers for early disease diagnosis and prognosis assessment (4). The work conducted in BSL is crucial for protecting human health and the environment, particularly when dealing with high-risk pathogens. However, BSL personnel face significant occupational stress from multiple sources, primarily due to their complex responsibilities and stringent biosafety regulations. They must manage various high-risk tasks while adhering strictly to biosafety standards, which poses challenges to their mental and physical health. Brandon Djukic’s research reveals a continuous rise in burnout among laboratory technicians. Since 2010, stress levels have significantly increased, with a nearly 2018% rise, while emotional exhaustion has risen by 14%. This trend highlights the potential impact of the work environment on the mental health of technicians and underscores the urgent need for attention (5).

In modern society, stress is widely recognized as a significant factor affecting psychological health. Workplace stress is nearly unavoidable due to the demands of the work environment. Across various professional groups, such as teachers (6), nurses (7), and doctors (8), there is a documented positive correlation between occupational stress and burnout. Occupational burnout is a condition resulting from factors such as job stress, which leads to changes in psychological and health states that deviate from normal functioning (9). Maslach describes burnout as a multidimensional state characterized by emotional exhaustion, depersonalization, and reduced personal accomplishment (10). Burnout not only negatively impacts workers’ mental and physical health but also severely impairs their work performance (11). Research indicates that severe burnout is closely related to sleep quality, with dissatisfaction with sleep patterns linked to emotional exhaustion and depersonalization among healthcare workers (12). Due to the unique nature of laboratory work, including high stress and irregular working hours, BSL (Biosafety Level) personnel are often at risk of diminished sleep quality. Poor sleep can lead to fatigue, reduced concentration, and slower reaction times, thereby negatively affecting work efficiency and quality.

At the 12th meeting of the Central Committee for Comprehensive Deepening Reform held in February 2020, General Secretary Xi Jinping explicitly stated that biosafety should be incorporated into the national security system. He emphasized the systematic planning of national biosafety risk prevention and control, as well as the development of a governance system, to comprehensively enhance the country’s biosafety governance capabilities. This policy addresses the weaknesses in the national biosafety system, aligns with public sentiment and social concerns, and plays a significant role in maintaining world peace. However, despite the gradual strengthening of biosafety measures, infections in biological laboratories continue to occur occasionally. According to relevant studies, these laboratory infection incidents are largely caused by human factors (13, 14). For instance, when laboratory staff encounter events such as infectious liquid spills or needle punctures, improper handling measures or irregular operations can potentially increase the risk of inhalation or contact infections (15).

Since the 19th century, incidents of laboratory-acquired infections have been frequently reported. Reports on accidents involving human pathogens and toxins in Canadian laboratories from 2016 to 2022 indicate that the root causes of exposure incidents are primarily related to standard operating procedures (n=211, 24%), human factors (n=183, 21%), and equipment issues (n=114, 13%) (16) or instance, in 1956, a laboratory in the former Soviet Union experienced an incident where nine ampoules containing Venezuelan equine encephalitis virus-infected mouse brains were broken. The incident was not managed according to the appropriate accident response protocols, resulting in 24 staff members were infected. In 1961, a laboratory in Moscow engaged in hemorrhagic fever research accidentally exposed wild mice from an epidemic area to the indoor environment due to operational errors, leading to aerosolized hemorrhagic fever virus contamination of the air and causing 113 laboratory staff infections and illnesses. Between 2003 and 2004, Singapore and Taiwan experienced laboratory leaks of Severe Acute Respiratory Syndrome (SARS) virus, leading to infections among laboratory personnel and sparking global concern over biosafety issues (17).

Therefore, the work capability of laboratory personnel is directly related to the safety of the laboratory. Evaluating staff work capability is crucial for preventing laboratory-acquired infections during public health emergencies. This study aims to investigate the levels of occupational burnout and sleep conditions among BSL personnel in various regions of Xinjiang, and to explore the impact of psychological health on the work performance of BSL employees. By conducting this research, we seek to gain a comprehensive understanding of the occupational health status of BSL personnel, thereby providing scientific evidence and new insights for improving work efficiency and enhancing biosafety measures.

The main innovations of this study are reflected in three aspects: Firstly, this research investigates the occupational mental health status of biosafety laboratory personnel in Xinjiang, filling a gap in the existing literature regarding the psychological health of this specific occupational group, both domestically and internationally, and highlighting the mental health issues faced by workers in the biosafety field within this unique context. Second, the study explores the potential benefits of assessing the mental health of biosafety laboratory personnel, emphasizing the impact of mental health improvement on individual work performance and the overall safety of the laboratory, thereby providing theoretical support for the development of occupational health intervention measures. Finally, this research employs innovative methodologies, including structural equation modeling and additive interaction analysis, to delve into the effects of occupational burnout and sleep quality on work capacity. Through path analysis, it reveals the interaction effects between these two factors, offering new insights into understanding this complex relationship and serving as an important reference for future research in related fields.

## Methods

### Participants and procedure

In July 2022, this study conducted a cluster sampling survey of Biosafety Level (BSL) personnel in the Xinjiang region. The participants included staff from disease control centers, hospital laboratories, and nucleic acid testing facilities, covering four prefecture-level cities, five districts, and five autonomous prefectures in Xinjiang. Before the survey commenced, we provided participants with a detailed explanation of the study’s purpose and invited them to voluntarily complete the questionnaire via the “Wenjuanxing” platform^1^. Trained personnel with relevant medical and psychological knowledge offered centralized guidance to respondents through the platform. Prior to the survey, we clarified the objectives, content, and requirements of the study to the participants, ensuring their understanding of the questionnaire’s nature, which encompassed occupational mental health and work capability. This study emphasized that participation was entirely voluntary, and participants had the right to withdraw at any time without affecting their work or training in the laboratory. Furthermore, we assured participants that their personal information and responses would be kept strictly confidential and used solely for research purposes, with no disclosure to third parties. To ensure the validity of the data, we excluded questionnaires with a completion rate below 100%, as well as those containing irregular, patterned, or obviously fraudulent responses.

The reasons for selecting cluster sampling are as follows: First, representativeness. This study was conducted during a biosafety training period for biosafety laboratory personnel, which effectively covered participants from multiple laboratories across Xinjiang, ensuring demographic diversity and representativeness of the sample and reflecting the overall mental health and work capability status of the workforce. Second, efficiency. Cluster sampling is relatively time and resource-efficient, especially when dealing with a dispersed and numerous laboratory workforce. Collecting data during a centralized meeting allows for the rapid acquisition of a large number of valid samples. Lastly, environmental consistency. By conducting the survey in the same training session, we ensured that participants completed the questionnaires in similar contexts and situations, thereby reducing the interference of external variables on the results and enhancing the internal validity of the study.

The inclusion criteria were as follows: (1) current employees working in BSL environments; (2) Individuals aged over 18 years with more than 1 year of experience in Biosafety Level (BSL) work and without a history of psychoactive substance use; (3) individuals who understood the purpose of the study and voluntarily participated. A total of 1980 BSL personnel members were invited to complete the questionnaire, with 1933 responses actually received. Three responses with irregular answers were excluded. Ultimately, 1930 participants were included in the study, resulting in a response rate of 97.4%. The study was approved by the Ethics Committee of the Urumqi Center for Disease Control and Prevention (Approval No. 20201125).

### Measures

#### Respondent information investigation

The Respondent Information investigation included 14 demographic and job-related variables, including gender, the level of BSL, age, education level, working years, professional title, marital status, monthly income(in RMB), shift work, chronic diseases (hypertension, coronary heart disease, diabetes), exercise frequency (per week).

#### Job burnout investigation

The Maslach Burnout Inventory-General Survey (MBI-GS) was developed by Maslach and Jackson (10, 18) in 1981 to assess the degree of occupational burnout by measuring emotional exhaustion, depersonalization, and depersonalization in the work environment. This study uses the Chinese version of the MBI-GS scale, which consists of 15 items and covers three dimensions: emotional exhaustion (EE, five items), depersonalization (four items) and Reduced Personal Accomplishment (six items). Each item on the scale is measured using a 7-point Likert scale, ranging from 0 (“Never”) to 6 (“Always”). The total score is calculated as follows: Total Score = 0.4 × Mean Emotional Exhaustion (EE) + 0.3 × Mean Reduced Personal Accomplishment (CY) + 0.3 × Mean Reduced Personal Accomplishment (PA). A total score ranging from 0 to 1.49 indicates no burnout, 1.50 to 3.49 indicates mild to moderate burnout, and 3.50 to 6 indicates severe burnout (19, 20). The Cronbach’s alpha coefficient of the scale in this study is 0.833, indicating good reliability.

#### Sleep quality investigation

Pittsburgh Sleep Quality Index (PSQI) was developed by (21) to assess retrospective sleep quality and sleep disturbances over the past month. It comprises seven dimensions of sleep quality, covering the following aspects: including the subjective sleep quality, sleep latency, sleep continuity, habitual sleep efficiency, sleep disorders and hypnotic drugs, daytime function seven factors。The total score is the sum of the scores from the seven dimensions, ranging from 0 to 21. According to domestic standards (22), a cumulative score greater than 7 indicates the presence of sleep problems. In this study, the Cronbach’s alpha coefficient for this scale is 0.872, indicating good reliability.

Reasons for Choosing the Pittsburgh Sleep Quality Index (PSQI) as the Sleep Quality Assessment Tool in this study:

1. Wide Applicability: The PSQI is an internationally recognized tool for evaluating sleep quality, having undergone extensive empirical research that validates its reliability and validity. It effectively reflects the individual’s sleep status, enhancing the credibility and comparability of the results in this study (23, 24).
2. Comprehensive Evaluation: The PSQI extends beyond merely assessing sleep quality; it encompasses several related dimensions, including sleep latency, sleep duration, sleep efficiency, sleep disturbances, frequency of sedative use, and daytime functioning (25). This multidimensional evaluation approach provides a holistic perspective for exploring the relationship between occupational mental health and work capability.
3. Convenience: The PSQI questionnaire is structured in a clear and straightforward manner, making it suitable for rapid implementation in large-scale surveys (26). This facilitates the efficient collection of substantial and valid data, aligning well with the design requirements of this study.
4. Cultural Adaptability: The PSQI has been validated across various cultural contexts and is applicable to diverse populations (27–29), making it particularly suitable for use among personnel in biosafety laboratories in the Xinjiang region.

In addition to the PSQI, several other commonly used assessment tools for sleep disorders exist, such as:

1. Hamilton Anxiety Scale (HAM-A): This tool is used to evaluate individual anxiety symptoms and their impact on sleep (30).
2. Epworth Sleepiness Scale (ESS): Primarily aimed at assessing the degree of daytime sleepiness, this scale indirectly reflects nighttime sleep quality (31).
3. Stanford Sleepiness Scale (SSS): This scale evaluates individuals’ sleep habits and related issues, making it suitable for a deeper understanding of the specific manifestations of sleep disorders (32).

#### Work ability investigation

The Work Ability Index (WAI) is a tool used in clinical occupational health and research to assess work ability during health examinations and workplace surveys (33). This scale includes a total of 7 items covering physiological, psychological, and disease-related aspects, with a total of 10 questions. The questionnaire consists of seven parts (part, score range), which are the current work ability compared with the lifetime best (part 1, 0–10), work ability related to professional needs (part 2, 2–10), the number of current diseases diagnosed by doctors (part 3, 1–7), estimated work disabilities caused by diseases (part 4, 1–6), the sick leave in the past year (part 5, 1–5), the prediction of working ability in 2 years from now (part 6,1,4 or 7), and mental resources (part 7, 1–4). The score of WAI was obtained by adding the scores of seven parts, which ranged from 7 to 49. The total scores of WAI were divided into four grades: poor (7–27), medium (28–36), good (37–43), and excellent (44–49). In order to facilitate the matching analysis of participants with PSA method, this study classified the good and excellent working ability as good (37–49 points), and the poor and medium working ability as poor (7–36 points). In this study, the Cronbach’s alpha coefficient for this scale is 0.666.

In this study, the reasons for choosing the Work Ability Index (WAI) as the assessment tool for work ability primarily include the following points:

1. Comprehensive Assessment: The WAI is an integrative scale that evaluates an individual’s work ability from multiple dimensions, including cognitive, emotional, and social capacities (34). This comprehensive assessment approach facilitates a thorough understanding of the work ability status of personnel in biosafety laboratories.
2. Rigorous Structure: The WAI was developed collaboratively by multiple experts and has undergone rigorous testing and validation, demonstrating high reliability and validity (35). This ensures the reliability and accuracy of the assessment results.
3. Practicality: The design of the WAI scale is straightforward, easy to understand, and use, making it particularly suitable for completing large-scale survey assessments within a short period. This aligns with the study’s need for efficient and rapid data collection (36).
4. Cultural Adaptability: The WAI has been widely applied across various countries and regions and is adaptable to different cultural backgrounds (37). Given that personnel in biosafety laboratories in the Xinjiang region may possess specific cultural characteristics, the choice of WAI better accommodates the assessment needs of this group.

In addition, there are several other commonly used methods for evaluating work ability, such as:

1. Holland’s Self-Directed Search (SDS): The tool is widely used for career planning, job counseling, and professional development, helping individuals identify career paths that are suitable for them (38).
2. Myers-Briggs Type Indicator (MBTI): MBTI is widely used in team building, career development, leadership training, and interpersonal communication, helping individuals identify and enhance their work abilities (39).
3. Career Adapt-Ability Scale (CAAS): It is commonly used in career counseling, educational assessment, and organizational human resource management, helping individuals identify their career development needs and formulate corresponding action plans. At the same time, this scale also assists organizations in evaluating employees’ adaptability, thereby optimizing team structure and employee training (40).

Although multiple assessment tools are available, the WAI has emerged as the preferred choice for this study due to its comprehensiveness, rigorous structure, practicality, and cultural adaptability. It effectively aids in the analysis of the relationship between the occupational mental health status of biosafety laboratory personnel and their work ability.

### Statistical analysis

Data analysis was conducted using R Studio (version 4.2.2). Descriptive statistics included frequencies (N) and proportions (%). Measurement data were summarized using means ± standard deviations (x ±s−). Comparisons between two independent group means were performed using independent samples t-tests, while comparisons among multiple group means were conducted using one-way ANOVA. Logistic regression was employed to further analyze factors affecting work ability among BSL employees, with job burnout and sleep quality included in the model to assess their interaction effects. The interaction effect was calculated using the Excel table developed by Andersson (41), which provided odds ratios (OR) and 95% confidence intervals for job burnout, sleep quality, and the coexistence of both factors. Additionally, the evaluation metrics for the interaction included the Relative Excess Risk due to Interaction (RERI), Attributable Proportion due to Interaction (AP), and the Synergy Index (S). An interaction was considered absent if the confidence intervals for RERI and AP included 0, and for S included 1 (42). Lastly, structural equation modeling was performed using AMOS 21.0 to analyze the path relationships between job burnout, sleep quality, and work ability among BSL employees. Model evaluation and correction were carried out using the Bollen-Stine *p*-value correction.

## Results

### Characteristics

Among the 1,930 BSL employees, 1,450 were female, accounting for 75.1% of the total workforce. There were 1,651 employees at the second level, representing 85.5% of the total. Employees under the age of 30 numbered 996, constituting 51.6% of the workforce. The numbers of employees with associate and bachelor’s degrees were 869 and 891, respectively, corresponding to 45.0 and 46.2%. Detailed data are presented in Table 1.

**Table 1:** Participant characteristics.

Index	Groups	*N* (%)
Sex	Male	480 (24.9)
	Female	1,450 (75.1)
The level of BSL	One	220 (11.4)
	Two	1,651 (85.5)
	Three	59 (3.1)
Age	18 ~ <30	996 (51.6)
	30 ~ 39	631 (32.7)
	40 ~ 49	216 (11.2)
	≥50	87 (4.5)
Education	Vocational or Below	112 (5.8)
	Associate Degree	869 (45)
	Bachelor’s Degree	891 (46.2)
	Graduate or Above	58 (3)
Working years	≤5	912 (47.3)
	6 ~ 10	429 (22.2)
	11 ~ 20	326 (16.9)
	21 ~ 30	190 (9.8)
	≥31	73 (3.8)
Professional title	Primary	1,483 (76.8)
	Middle	319 (16.5)
	Senior	128 (6.6)
Marital status	Unmarried	759 (39.3)
	Married	1,171 (60.7)
Monthly income (In RMB)	≤4,000	859 (44.5)
	>4,000	1,071 (55.5)
Hypertension	Yes	76 (3.9)
	No	1854 (96.1)
Coronary heart disease	Yes	35 (1.8)
	No	1895 (98.2)
Diabetes	Yes	24 (1.2)
	No	1906 (98.8)
Shift assignment	Day shift	588 (30.5)
	Night shift	1,342 (69.5)
	Never	613 (31.8)
Exercise (peer weak)	1 ~ 2 times	886 (45.9)
	3 ~ 4 times	303 (15.7)
	5 times or above	128 (6.6)
Job burnout	Yes	1,300 (67.4)
	No	630 (32.6)
Sleep quality	Good sleep quality	1,179 (61.1)
	Sleep disorder	751 (38.9)

### Survey on job burnout, sleep quality, and work ability among BSL employees

67.4% of BSL employees exhibit signs of job burnout. Sleep disorders affect 38.9% of employees, with an average sleep quality score of 10.58 ± 2.41. The work ability index for BSL employees is 38.83 ± 6.43. Among the four levels of work ability, the majority of employees are categorized as having moderate (28.1%) and good (41.2%) work ability. Fewer employees fall into the poor category (5.2%), while 25.5% are classified as having excellent work ability. This indicates that most oil workers have moderate to good work ability, with a smaller proportion exhibiting very poor work ability. Detailed data can be found in Table 2.

**Table 2:** Scores of occupational burnout, sleep quality, and work ability.

Variables	Scores	*N* (%)
Job burnout	1.87 ± 0.86	1930 (100.0)
No burnout	0.94 ± 0.42	630 (32.6)
Moderate burnout	2.22 ± 0.46	1,235 (64.0)
Severe burnout	4.10 ± 0.50	65 (3.4)
Sleep quality	6.78 ± 3.72	1930 (100.0)
Good sleep quality	4.36 ± 1.99	1,179 (61.1)
Sleep disorder	10.58 ± 2.41	751 (38.9)
Work ability	38.83 ± 6.43	1930 (100.0)
Poor	23.03 ± 3.66	100 (5.2)
Medium	33.00 ± 2.43	543 (28.1)
Good	40.33 ± 1.95	795 (41.2)
Excellent	46.04 ± 1.63	492 (25.5)

### Comparison of WAI score among different sub-groups

The work ability index among BSL employees shows significant statistical differences based on gender, age, educational level, years of service, job title, marital status, monthly income, chronic conditions (such as hypertension, coronary heart disease, diabetes), and frequency of exercise (all *p*-values <0.05). Specifically: males have a higher work ability index than females; work ability decreases with age; a higher level of education is associated with a higher work ability index; increasing years of service are linked to a lower work ability index; employees with lower job titles have a higher work ability index; those who exercise weekly have a significantly higher work ability index compared to those who do not exercise; and unmarried employees, those without chronic diseases, those without job burnout, and those with good sleep quality have a higher work ability index compared to other groups. Detailed data are presented in Table 3.

**Table 3:** Comparison of WAI score among different sub-groups.

Index	Groups	WAI scores	t/*F*-value	*P*-value
Sex	Male	39.71 ± 6.70	3.494	<0.01
	Female	38.53 ± 6.31		
The level of BSL	One	38.18 ± 7.06	2.988	0.051
	Two	38.96 ± 6.30		
	Three	37.39 ± 7.20		
Age	18 ~ <30	39.38 ± 6.50	11.908	<0.01
	30 ~ 39	38.82 ± 6.07		
	40 ~ 49	37.47 ± 6.33		
	≥50	35.90 ± 7.12		
Education	Vocational or Below	34.62 ± 7.96	18.037	<0.01
	Associate Degree	38.95 ± 6.70		
	Bachelor’s Degree	39.16 ± 5.80		
	Graduate or Above	39.98 ± 5.16		
Working years	≤5	39.54 ± 6.40	13.197	<0.01
	6 ~ 10	39.41 ± 5.90		
	11 ~ 20	37.53 ± 6.37		
	21 ~ 30	37.37 ± 6.66		
	≥31	36.05 ± 7.25		
Professional title	Primary	39.16 ± 6.36	11.542	<0.01
	Middle	38.18 ± 6.38		
	Senior	36.58 ± 6.73		
Marital status	Unmarried	39.34 ± 6.31	2.807	<0.01
	Married	38.50 ± 6.48		
Monthly income (In RMB)	≤4,000	38.98 ± 6.51	0.953	0.341
	>4,000	38.70 ± 6.36		
Hypertension	Yes	34.24 ± 7.13	−6.421	<0.01
	No	39.02 ± 6.33		
Coronary heart disease	Yes	31.17 ± 6.27	−7.208	<0.01
	No	38.97 ± 6.34		
Diabetes	Yes	32.96 ± 7.33	−4.526	<0.01
	No	38.90 ± 6.38		
Shift assignment	Day shift	39.01 ± 6.30	0.837	0.403
	Night shift	38.75 ± 6.48		
	Never	37.18 ± 6.02	21.767	<0.01
Exercise (peer weak)	1 ~ 2 times	39.36 ± 6.37		
	3 ~ 4 times	39.89 ± 6.77		
	5 times or above	40.52 ± 6.36		
Job burnout	Yes	36.78 ± 6.22	25.256	<0.01
	No	43.05 ± 4.49		
Sleep quality	Good sleep quality	40.84 ± 5.63	18.284	<0.01
	Sleep disorder	35.66 ± 6.33		

### Multivariate logistic regression analysis of work ability among BSL personnel

Binary logistic regression analysis was conducted with work ability (where good and excellent = 0, poor and medium = 1) as the dependent variable and all statistical variables as independent variables. The results indicated that educational level, years of service, chronic diseases (hypertension, coronary heart disease), weekly exercise frequency, occupational burnout, and sleep quality were included in the regression model. Specifically, the findings are as follows: Educational levels of college (OR = 0.523, 95% CI: 0.322–0.848), undergraduate (OR = 0.393, 95% CI: 0.239–0.646), and postgraduate or higher (OR = 0.204, 95% CI: 0.086–0.486) serve as protective factors for work ability. A years of service range of 21–30 years (OR = 2.224, 95% CI: 1.288–3.839) is associated with poorer work ability. Hypertension (OR = 2.015, 95% CI: 1.105–3.674), coronary heart disease (OR = 3.404, 95% CI: 1.208–9.588), and occupational burnout (OR = 6.468, 95% CI: 4.728–8.848) are identified as risk factors for work ability. Weekly exercise frequencies of 1–2 times (OR = 0.676, 95% CI: 0.526–0.869), 3–4 times (OR = 0.626, 95% CI: 0.438–0.895), and more than 5 times (OR = 0.531, 95% CI: 0.319–0.885) are associated with better work ability. Sleep quality (OR = 3.318, 95% CI: 2.646–4.161) is also a significant factor affecting work ability. The results suggest that higher educational levels and more frequent exercise are protective factors for BSL employees’ work ability. Specifically, higher education levels and increased weekly exercise frequency are linked to better work ability. Conversely, years of service, chronic diseases, occupational burnout, and sleep disorders are risk factors for work ability, with years of service between 21 and 30 years, hypertension, coronary heart disease, occupational burnout, and sleep disorders all contributing to reduced work ability among BSL employees. Detailed data are presented in Table 4.

**Table 4:** Multivariate logistic regression analysis of work ability.

Variable	Comparison group	*B*	SE	Wald χ^2^	*p*-value	Odd ratio (95%CI)
Education	Vocational or Below			19.397	<0.01	
	Associate Degree	−0.649	0.247	6.895	<0.01	0.523 (0.322 ~ 0.848)
	Bachelor’s Degree	−0.934	0.253	13.602	<0.01	0.393 (0.239 ~ 0.646)
	Graduate or Above	−1.588	0.442	12.916	<0.01	0.204 (0.086 ~ 0.486)
Working years	≤5			16.011	<0.01	
	21 ~ 30	0.799	0.279	8.228	<0.01	2.224 (1.288 ~ 3.839)
Hypertension	Yes	0.701	0.306	5.225	<0.05	2.015 (1.105 ~ 3.674)
Coronary heart disease	Yes	1.225	0.528	5.372	<0.05	3.404 (1.208 ~ 9.588)
Exercise (peer weak)	Never			13.503	<0.01	
	1 ~ 2 times	−0.391	0.128	9.357	<0.01	0.676 (0.526 ~ 0.869)
	3 ~ 4 times	−0.468	0.182	6.585	<0.05	0.626 (0.438 ~ 0.895)
	5 times or above	−0.632	0.260	5.912	<0.05	0.531 (0.319 ~ 0.885)
Job burnout	Yes	1.867	0.160	136.349	<0.01	6.468 (4.728 ~ 8.848)
Sleep quality	Sleep disorder	1.199	0.115	107.856	<0.01	3.318 (2.646 ~ 4.161)

### The interaction effect of job burnout and sleep quality on the impact on work ability

Logistic regression analysis was conducted with work ability (where good and excellent = 0, poor and medium = 1) as the dependent variable, using employees with no burnout and good sleep as the reference group. The results revealed that BSL employees with only occupational burnout had a 3.301-fold higher risk of impaired work ability compared to those without burnout (OR = 3.301, 95% CI: 1.856–5.871). BSL employees with only sleep disorders faced a 6.452-fold higher risk of impaired work ability compared to those with good sleep (OR = 6.452, 95% CI: 4.317–9.644). Employees with both occupational burnout and sleep disorders had a 21.430-fold higher risk of impaired work ability compared to those with neither condition (OR = 21.430, 95% CI: 14.300–32.118). Interaction analysis showed an S value of 2.635 (95% CI: 1.932–3.594), indicating that the risk of impaired work ability for BSL employees with both occupational burnout and sleep disorders is 2.635 times the sum of the risks associated with each condition individually. The RERI value was 12.676, and the API (%) value was 59.152%, demonstrating a significant additive interaction between the two conditions. Detailed data can be found in Table 5.

**Table 5:** The interaction effect of job burnout and sleep quality on the impact on work ability.

Variable	Variable	Additive interaction analysis				
		[Work ability of (poor, medium)] (*n*, %)	Odds ratio (95%CI)	RERI (95%CI)	AP (95%CI)	S (95%CI)
Job burnout	Sleep quality					
No	No	33 (6.48%)	1	12.676 (6.668 ~ 18.684)	59.152% (47.796% ~ 70.508%)	2.635 (1.932 ~ 3.594)
No	Yes	27 (22.31%)	3.301 (1.856 ~ 5.871)			
Yes	No	200 (29.85%)	6.452 (4.317 ~ 9.644)			
Yes	Yes	383 (60.79%)	21.430 (14.300 ~ 32.118)			

### Structural equation model of career burnout, sleep quality, and work ability

#### Structural equation model fit results

**Figure 1:**
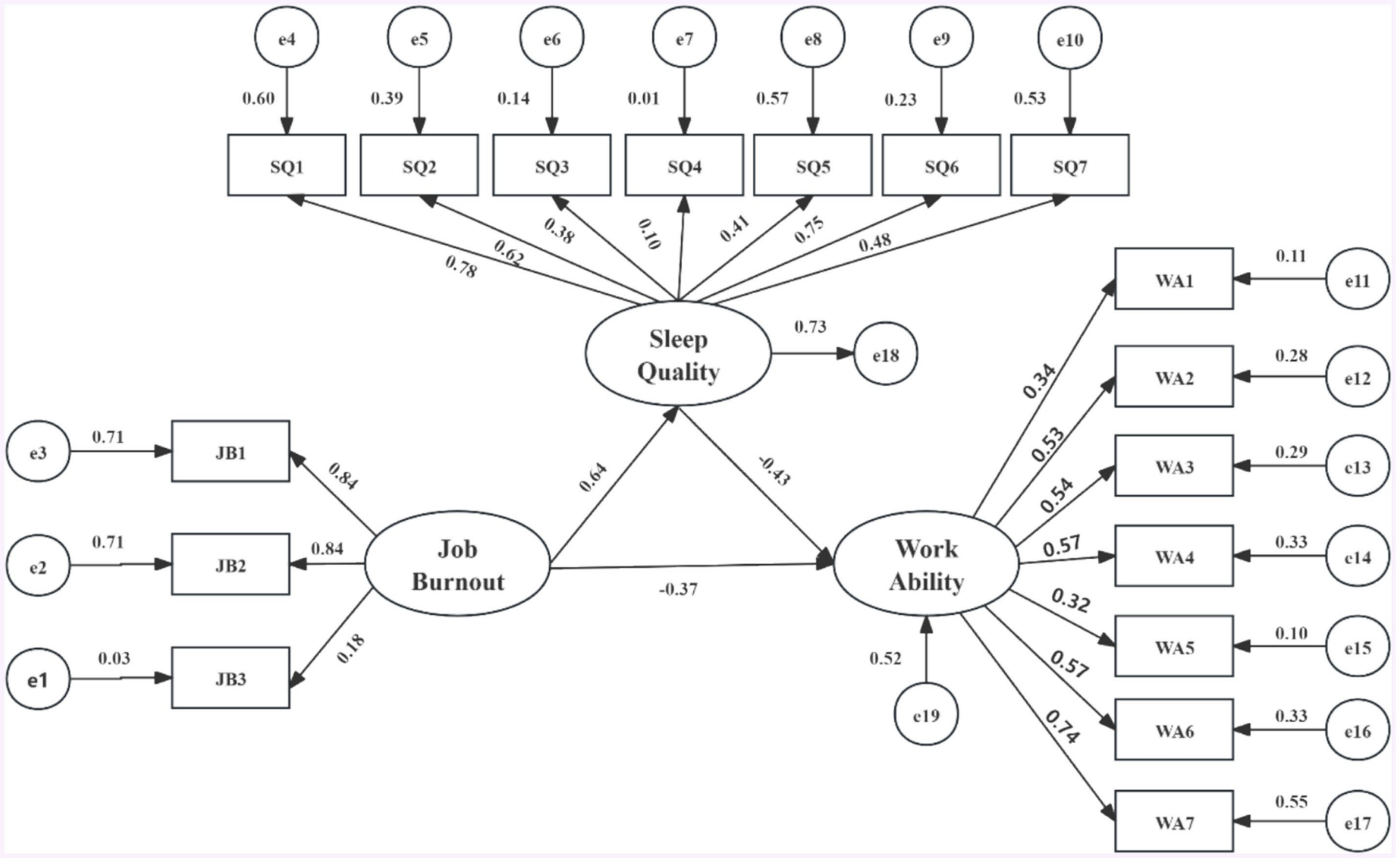
Structural equation model of career burnout, sleep quality, and work ability. Structural equation modeling results for the relationship between burnout, sleep quality, and work ability. All coefficients in the figure are standardized and significant at the 0.05 level. JB1, emotional exhaustion; JB2, depersonalization; JB3, reduced personal accomplishment; SQ1, the subjective sleep quality; SQ2, sleep latency; SQ3, sleep continuity; SQ4, habitual sleep efficiency; SQ5, sleep disorders; SQ6, hypnotic drugs; SQ7, daytime function seven factors; WA1, the lifetime best; WA2, work ability related to professional needs; WA3, the number of current diseases diagnosed by doctors; WA4, estimated work disabilities caused by diseases; WA5, the sick leave in the past year; WA6, the prediction of working ability in 2 years from now; WA7, and mental resources.

This study constructed a structural equation model with sleep quality and work ability as endogenous variables and occupational burnout as an exogenous variable (see Figure 1). The evaluation indices for the fit of the structural equation model include: absolute fit indices such as the chi-square value (χ^2^), goodness-of-fit index (GFI), adjusted goodness-of-fit index (AGFI), and root mean square error of approximation (RMSEA); and incremental fit indices such as the normed fit index (NFI), incremental fit index (IFI), and comparative fit index (CFI) (43). The initial model’s fit indices did not meet the standards, and the model analysis showed a *p*-value of 0, indicating poor model fit. Typically, a significant p-value may be due to two reasons: either a very large sample size or poor model fit (44). To address this issue, the study employed a bootstrap procedure with 5,000 repetitions. The results of the Bollen-Stine bootstrap test showed: (1) The model fit better in 5000 bootstrap samples; (2) It fit about equally well in 0 bootstrap samples; (3) It fit worse or failed to fit in 0 bootstrap samples; (4) Testing the null hypothesis that the model is correct, *p* = 0. These results indicate that after 5,000 bootstrap replications, the model exhibited good fit in 5000 instances and poor fit in 0 instances. Therefore, we can reject the null hypothesis, suggesting that the significant *p*-value was due to the large sample size rather than poor model fit. Consequently, this study evaluated and adjusted the model using Bollen-Stine *p*-value correction, and the corrected indices all met the standards. The fit indices before and after correction are presented in Table 6.

**Table 6:** Fit indices of structural equation model.

Model fit indices	ML	Bollen-Stine *P*-value	Criteria
χ^2^/df	19.073	1.23	<3.0
RMSEA	0.10	0.01	<0.08
GFI	0.88	0.99	>0.90
AGFI	0.84	0.98	>0.90
NFI	0.78	0.99	>0.90
TLI	0.76	1.00	>0.90
IFI	0.79	1.00	>0.90
CFI	0.79	1.00	>0.90

ML, maximum likelihood estimation.

### Path coefficients between job burnout, sleep quality, and work ability

The results of the structural equation model analysis indicate that both occupational burnout and sleep quality have direct effects on work ability. Specifically, higher levels of occupational burnout and poorer sleep quality are associated with lower work ability, with standardized path coefficients (*β*) of −0.37 and −0.43, respectively. Additionally, occupational burnout also indirectly affects work ability through sleep quality. As occupational burnout increases and sleep quality declines, the standardized path coefficient (β) is 0.64, leading to an indirect effect of occupational burnout on work ability of −0.28 (0.64 × −0.43). For further details, please refer to Figure 1 and Table 7.

**Table 7:** Structural equation model path regression coefficients.

Path	Unstandardized regression coefficients	SE	CR	*P*-value	Standardized regression coefficients
Job burnout → Sleep quality	1.547	0.225	6.87	<0.01	0.637
Sleep quality → Work ability	−0.461	0.049	−9.447	<0.01	−0.426
Job burnout → Work ability	0.969	0.165	−5.86	<0.01	−0.368

## Discussion

### Elevated rates of occupational burnout and sleep disorders among BSL employees

In 2019, the World Health Organization (WHO) released the 11th edition of the International Classification of Diseases, which included burnout as a medical condition, defining it as a syndrome resulting from chronic workplace stress that has not been successfully managed, characterized by feelings of exhaustion, cynicism, and reduced professional efficacy (45). Research indicates that prolonged job stress and persistent emotional and mental fatigue not only lead to mental health issues such as anxiety, depression, and emotional instability, but are also associated with physical health problems including sleep disorders, digestive system diseases, and cardiovascular diseases (46–48). Furthermore, long-term burnout can have negative impacts on organizations and society, such as increased employee turnover intentions, leading to higher recruitment and training costs (49). At the societal level, prolonged burnout may result in escalated healthcare costs and social welfare expenditures, ultimately affecting economic development and social stability. As professionals handling highly infectious biological materials and pathogens in laboratory settings, Biosafety Level (BSL) personnel play a crucial role in preventing disease transmission and protecting human and environmental health and safety. Therefore, their physical and mental well-being deserves utmost attention.

This study shows that employees from several biosafety laboratories (BSL) in Xinjiang reported significant levels of occupational burnout and sleep quality issues. These laboratories primarily engage in research and testing related to pathogenic microorganisms. Despite having implemented biosafety measures, some employees still feel at risk of pathogen exposure. Some participants in this study come from county-level health departments, where there are issues such as aging or damaged key facilities, including biosafety cabinets and waste disposal equipment, which may increase the risk of pathogen leakage. Additionally, some laboratory personnel in health departments have not strictly adhered to standards for personal protection, such as masks, gloves, goggles, and isolation gowns that do not meet usage standards. These factors contribute to the risk of pathogen exposure. Our study investigated burnout among 1930 BSL personnel in Xinjiang and found a burnout detection rate of 67.4%, which is significantly higher than the results of other relevant studies; for example, in a systematic review encompassing 161 studies, only 47% of 341,014 healthcare workers worldwide reported experiencing burnout. This significant difference highlights the unique pressures and challenges faced by personnel in the Xinjiang Biosafety Laboratory (50). This phenomenon may be attributed to the stress caused by the risk of COVID-19 infection. Related studies have shown that the incidence of burnout among healthcare workers during the COVID-19 pandemic was higher than in the past, which is associated with the increased psychological burden on medical system workers due to high virus exposure risk and intense workload (51). The subjects of this study include workers who come into contact with biological samples, such as virology laboratory personnel, nucleic acid testing personnel, and hospital laboratory personnel. They face high virus exposure due to their occupational characteristics, increasing the risk of mental health issues.

Sleep profoundly influences various aspects of the body, playing a crucial role in physical and mental health. Almost every country reports complaints about sleep disorders (or poor sleep quality) (52). Untreated sleep disorders can pose life-threatening risks; they are not only the result of medical conditions but also significant drivers of other diseases (53). Poor sleep quality significantly affects daytime performance, including social and work functions (54), increases the risks of occupational and traffic accidents (55), and diminishes quality of life and overall health (56). Numerous studies suggest links between sleep disorders and attention deficit, depression, anxiety, impaired cognitive function, and neurocognitive dysfunction (57, 58). Therefore, evaluating sleep quality is vital in epidemiological and clinical research. This survey found an overall sleep disorder detection rate of 38.9% among employees at BSL in Xinjiang. According to the “China Sleep Index,” the sleep quality scores of medical workers have continued to rank second from the bottom for several years, with a sleep disorder incidence rate as high as 39.9%, which is consistent with the findings of this study. Sleep issues such as insufficiency and poor sleep quality directly reduce the operational efficiency and work ability of BSL personnel, and in severe cases, even threaten their lives. Studies by Pappa S and Ntella V, among others, have shown that during the COVID-19 pandemic, frontline healthcare workers faced anxiety and high stress levels, which resulted in decreased sleep quality (59). As virus detection personnel, BSL employees undertake heavy workloads and are concerned about highly infectious viral samples, leading to considerable psychological stress that may impair their sleep quality. The survey also found that 69.9% of BSL employees required shifts, which is a primary factor affecting their sleep quality. Night shifts disrupt the circadian rhythm, leading to confusion in the sleep–wake cycle. Moreover, exposure to bright environments, especially blue light, suppresses melatonin secretion, compromising sleep quality. Research indicates that individuals who work night shifts for extended periods have lower melatonin levels compared to those working fixed day shifts (60).

### Factors reducing work ability among BSL employees: (female, older age, low education level, short work experience, low professional title, married, low income, hypertension, diabetes, coronary heart disease, low exercise frequency)

Based on the findings of this study, the work capability level of BSL employees in Xinjiang is relatively high, with a detection rate of 66.7%, slightly surpassing the results of a French survey involving 4,306 medical professionals (65.5%) (61). This suggests that the overall work capability of Xinjiang BSL personnel is satisfactory. This positive outcome may be attributed to the generally high educational attainment of the study participants, who predominantly hold college and bachelor’s degrees, and the fact that they are primarily young individuals under the age of 40. These characteristics likely contribute to their enhanced stress resistance and adaptability compared to the general population. Additionally, the survey participants, being BSL personnel during the COVID-19 pandemic, play a critical role in the control and progression of the outbreak. Consequently, due to the significant responsibilities they bear, their work capability requirements are notably higher than in the past (62).

The study found that male employees generally exhibit better work capabilities than female employees, which may be attributed to advantages in psychological resilience and stress tolerance among men. This disparity is particularly evident during the COVID-19 pandemic when the extensive virus sample testing highlights men’s physical endurance (63). Additionally, work capability tends to decline with age and years of service. This decline could be due to the gradual deterioration of physical functions such as strength, endurance, and reaction time with age. Furthermore, long-term engagement in the same job can lead to professional burnout, reducing enthusiasm and motivation, which subsequently affects work performance. Topa et al. (64) indicated that although employees may attempt to enhance their job satisfaction and engagement by adjusting their work tasks and methods, these adjustments may, over time, actually lead to a decrease in their sense of engagement. This can result in older employees, compared to their younger counterparts, being less attracted to developmental goals and high levels of effort, making them more vulnerable to feelings of burnout.

Among BSL employees, those with higher professional titles generally have lower work capability indices. Employees with junior titles exhibit better work capabilities compared to those with intermediate or senior titles. This could be due to the fact that promotions often involve additional management and leadership responsibilities, requiring more time and effort on administrative tasks and reducing opportunities for hands-on scientific research, thereby leading to a relative decline in work capability. The study also found that unmarried individuals have better work capabilities. Research by Giacomo Garzaro (65) indicates that single workers exhibit significantly higher work capabilities than their married counterparts. This could be because single employees are typically younger, have lower BMI, and enga0067e in more leisure exercise weekly. In contrast, married individuals face higher financial and psychological pressures, which may negatively impact their work capability. Moreover, multiple regression analysis results indicate that educational level has a protective effect on BSL employees’ work capability. Higher educational attainment is associated with stronger work capability. This is because individuals with higher education typically undergo more systematic and in-depth learning, possess broader and deeper knowledge, and are better equipped to understand and apply work-related theories, techniques, and methods, thereby enhancing work efficiency and quality.

Multifactorial analysis results indicate that the frequency of weekly exercise positively influences the work capacity of BSL employees. Moderate exercise can relieve physical and mental tension and promote the release of neurotransmitters such as dopamine, endorphins, and serotonin, which contribute to improved mood (66). Furthermore, moderate physical activity is associated with enhanced sleep quality, thereby increasing daily work efficiency. This study found that BSL personnel with chronic illnesses exhibit lower work efficiency. This can be explained by biopsychosocial theories, which suggest that chronic illnesses lead to physical instability, subsequently affecting cognitive abilities and work performance. Additionally, chronic conditions may impair the functioning of the brain and nervous system, further impacting individuals’ ability to cope with work tasks (67). A cohort study involving 38,470 participants with chronic diseases revealed a strong negative correlation between cardiovascular diseases and type 2 diabetes (DM2) and work efficiency in mid- to late-career stages (68). The study also demonstrated that occupational burnout significantly negatively affects the work capabilities of BSL personnel. A survey of 3,030 nurses found that 64.3% reported experiencing burnout, with 36.5% planning to leave their hospital within a year. A significant correlation exists between high levels of burnout and the intention to leave (69). Moreover, the results indicate that sleep disorders are a crucial factor impacting the work efficiency of BSL personnel. According to perspectives from sleep medicine, sleep disorders can affect individuals’ alertness and attention, subsequently influencing work efficiency and the safety of laboratory operations.

### Job burnout and sleep disorders reduce the work ability of BSL personnel

This study investigated the additive interactions of influencing factors using a logistic regression model. The results revealed that the risk of reduced work capacity is 21.430 times higher in individuals experiencing both occupational burnout and sleep disorders compared to the reference group (those without burnout and with good sleep quality). This finding indicates a significant additive interaction between these factors. According to the two-factor theory, job satisfaction or dissatisfaction consists of two independent but interacting factors: job content and job environment (70). These factors interact to shape employees’ work experiences. In BSL, occupational burnout and sleep quality can be seen as the “satisfaction” and “dissatisfaction” factors in this theory. Occupational burnout pertains to job content, such as work stress, workload, and mental strain, while sleep quality relates to job environment factors, such as work schedules and environmental impacts. Burnout affects employees’ cognitive and decision-making abilities, thereby impacting work quality and efficiency. Poor sleep quality, on the other hand, affects mood, physical condition, and concentration, leading to decreased work efficiency and quality, and even increasing the risk of accidents. A structural equation model was established to further explore the pathways between occupational burnout, sleep quality, and work capacity. The results showed that sleep quality mediates the relationship between occupational burnout and work capacity. Studies have indicated that high levels of occupational burnout not only lead to sleep problems but also that poor sleep can exacerbate or trigger burnout (71). Brubaker et al. (72) suggested that health promotion should prioritize sleep quality as an early risk indicator, with interventions focusing on improving sleep to reduce the incidence of burnout. These findings highlight the close relationship between occupational burnout and sleep quality, and how both issues can severely impact the health and work capacity of professionals, significantly increasing the risk of workplace accidents.

Based on the findings of this study, occupational burnout and sleep disorders significantly affect the work capacity of biosafety laboratory (BSL) employees. The participants in this study come from multi-center laboratories across 106 counties in 4 prefecture-level cities, 5 regions, and 5 autonomous prefectures in Xinjiang, including relevant disease control and prevention centers, hospital laboratory departments, and other institutions. These laboratories primarily focus on the detection of pathogens causing infectious diseases and vaccine development. Among them, there are BSL-1, BSL-2, and BSL-3/4 laboratories. BSL-1 laboratories are mainly used for academic research on non-pathogenic microorganisms or other tissue cultures under basic experimental conditions; BSL-2 laboratories are primarily used for handling moderately hazardous pathogens, including those that cause local infections and inhalation infections; BSL-3/4 laboratories are mainly used for operating highly hazardous pathogens, such as Ebola virus and variola major virus. However, during the study, we found that some laboratories have inadequacies in protective measures against pathogen exposure, which may influence employees’ mental health and work capacity. The results from the structural equation modeling analysis suggest that reducing occupational burnout and improving sleep quality can enhance the work capacity of BSL employees in Xinjiang. To address these issues, relevant organizations should offer training on sleep health and management and promote healthy sleep practices, such as regular sleep schedules and manageable work intensity. Furthermore, while participants are required to undergo biosafety training upon employment to ensure they understand laboratory protocols and safety procedures, there is a need to enhance the specificity of these training programs to better equip employees in managing work-related challenges. Attention should also be given to employees’ mental health, with timely interventions for negative psychological conditions, as these are crucial for improving work capacity. Implementing these measures is essential for enhancing overall job performance and reducing occupational burnout.

## Limitations

This study has several limitations that warrant consideration. First, the use of a cross-sectional design limits the ability to establish causal relationships between occupational burnout, sleep quality, and work ability. Therefore, future longitudinal studies would be more beneficial in elucidating the dynamic relationships among these variables over time. Second, the reliance on self-reported measurement tools, such as the Maslach Burnout Inventory and the Pittsburgh Sleep Quality Index, may introduce response bias, as participants might overestimate or underestimate their levels of burnout and sleep quality. Additionally, this study is focused exclusively on biosafety laboratory staff in the Xinjiang region, which may restrict the generalizability of the findings to other regions or different types of laboratory environments. Consequently, future research should aim to include a broader population across various locations and laboratory settings to enhance the external validity of the results. Lastly, while this study identified significant interactions and mediating effects, other potential confounding factors, such as workload, health status, and individual coping strategies, were not thoroughly examined. Considering these factors in subsequent research may provide a more comprehensive understanding of the multidimensional variables influencing the work ability of biosafety laboratory personnel.

## Data Availability

The original contributions presented in the study are included in the article/supplementary material, further inquiries can be directed to the corresponding author.

## Footnotes

^1^ https://www.wjx.cn/vm/rX4XJXc.aspx#
